# Enzyme-Linked Immunosorbent Assay Characterization of Basal Variation and Heritability of Systemic Microfibrillar-Associated Protein 4

**DOI:** 10.1371/journal.pone.0082383

**Published:** 2013-12-04

**Authors:** Susanne Gjørup Sækmose, Anders Schlosser, René Holst, Sofie Lock Johansson, Helle Wulf-Johansson, Ida Tornøe, Jørgen Vestbo, Kirsten Ohm Kyvik, Torben Barington, Uffe Holmskov, Grith Lykke Sørensen

**Affiliations:** 1 Cardiovascular and Renal Research, Institute of Molecular Medicine, University of Southern Denmark, Odense, Denmark; 2 Department of Clinical Immunology, Region Sjaelland, Naestved Hospital, Naestved, Denmark; 3 Department of Clinical Immunology, Odense University Hospital, Odense, Denmark; 4 Danish Twin Registry and Danish Aging Research Centre, Institute of Public Health, University of Southern Denmark, Odense, Denmark; 5 Department of Respiratory Medicine, Odense University Hospital, Odense, Denmark; 6 Respiratory and Allergy Research Group, Manchester Academic Health Sciences Centre, University Hospital South Manchester NHD Foundation Trust, Manchester, United Kingdom; 7 Odense Patient data Explorative Network (OPEN), Odense University Hospital, Odense, Denmark; Karolinska Institutet, Sweden

## Abstract

**Background:**

Microfibrillar-associated protein 4 (MFAP4) is a systemic biomarker that is significantly elevated in samples from patients suffering from hepatic cirrhosis. The protein is generally localized to elastic fibers and other connective tissue fibers in the extracellular matrix (ECM), and variation in systemic MFAP4 (sMFAP4) has the potential to reflect diverse diseases with increased ECM turnover. Here, we aimed to validate an enzyme-linked immunosorbent assay (ELISA) for the measurement of sMFAP4 with an emphasis on the robustness of the assay. Moreover, we aimed to determine confounders influencing the basal sMFAP4 variability and the genetic contribution to the basal variation.

**Methods:**

The sandwich ELISA was based on two monoclonal anti-MFAP4 antibodies and was optimized and calibrated with a standard of recombinant MFAP4. The importance of pre-analytical sample handling was evaluated regarding sample tube type, time, and temperature conditions. The mean value structure and variance structure was determined in a twin cohort including 1,417 Danish twins (age 18-67 years) by mixed-effect linear regression modeling.

**Results:**

The practical working range of the sandwich ELISA was estimated to be 4-75 U/ml. The maximum intra- and inter-assay variation was estimated to be 8.7% and 6.6%, respectively. Sample handling and processing appeared to influence MFAP4 measurements only marginally. The average concentration of sMFAP4 in the serum was 18.9 ± 8.4 (SD) U/ml in the twin cohort (95% CI: 18.5-19.4, median sMFAP4 17.3 U/ml). The mean structure model was demonstrated to include waist-hip ratio, age, and cigarette smoking status in interactions with gender. A relatively low heritability of h^2^ = 0.24 was found after applying a model including additive genetic factors and shared and non-shared environmental factors.

**Conclusions:**

The described ELISA provides robust measures of the liver fibrosis marker sMFAP4. The low heritability and the relatively limited basal variation suggest that increased sMFAP4 reflects disease-induced processes.

## Introduction

Microfibrillar-associated protein 4 (MFAP4) is a 66-kDa homodimeric protein including a C-terminal fibrinogen-related domain and an N-terminal domain that is involved in disulfide-bridge cross-linking of the monomers into dimers. The gene encoding human MFAP4 was described more than 15 years ago and was identified as one of the genes deleted from chromosome 17 in Smith-Magenis Syndrome [1]. The porcine protein homologue MAGP-36 is demonstrated to possess an integrin interaction domain (RGD-motif) in the amino-terminal region, and immuno-electron microscopy showed that the protein is localized in the arterial adventitia specifically surrounding the elastin-associated microfibrils [2]. The bovine homologue is further shown to bind directly to elastin as well as type I collagen [3]. Likewise, the rat homologue MAGP-36 is demonstrated to be present at sites of high elastin content. MAGP-36 protein expression is highest in the aorta, whereas the expression of MAGP-36 mRNA is highest in lung and trachea [4].

The expression profile of human MFAP4 is not fully characterized. In humans, MFAP4 is reported to be highly expressed in tissues of high elastin content such as the wall of elastic arteries and the skin [5,6]. Moreover, MFAP4 is present as a soluble protein in lung washings and co-localizes with elastic fibers in the alveolar septae in the human lung [5].

The physiological role of MFAP4 remains largely unknown. MFAP4 is suggested to play a role in maintaining the integrity of the extracellular matrix (ECM) in organs of high tensile strength, such as the aorta [7]. Moreover, MFAP4 is suggested to participate in inflammatory processes in the lung [5,8]. Based on its localization in a segment of the proximal tubules in the kidney, a role in mannose transport is also suggested [4]. Recent data support a role for MFAP4 in tissue homeostasis. MFAP4 is localized in the periphery of elastic fibers in the skin and is suggested to be an essential component in microfibril development. UVB irradiation decreases the level of MFAP4, whereas over-expression of MFAP4 in a skin-xenografted mouse-model was demonstrated to protect from photodamage with reduced degradation of extracellular matrix proteins [9].

In general, remodeling of the extracellular matrix is important in tissue homeostasis, and the normal balance is disturbed in many pathological situations including fibrosis [10]. A large range of proteins, including extracellular matrix proteins and components, are relevant to consider when searching for biomarkers to detect developing pathology in tissue homeostasis, potentially leading to fibrosis. The main ECM substances are collagens and proteoglycans, and derived components such as hyaluronic acid can be measured in the peripheral blood. Various matrix metalloproteinases and their inhibitors have also been investigated as markers of ECM remodeling in a variety of pathologies [11-13].

Recently, a search for novel biomarkers in HCV-associated hepatic cirrhosis revealed MFAP4 to be increased in hepatic fibrosis [14]. MFAP4 was highly abundant in the cirrhotic septae, and furthermore, systemic MFAP4 (sMFAP4) significantly increased with progressive fibrosis stage, indicating that sMFAP4 may be a novel candidate systemic biomarker. High diagnostic accuracy for the prediction of non-diseased liver versus cirrhosis was found [14].

Here, the validation of an ELISA measuring human sMFAP4 is described with an emphasis on the robustness of the assay. This description includes an analysis of pre-analytical sample handling procedures addressing time from sample withdrawal to centrifugation, temperature conditions, and the type of sample tube used. Moreover, we set out to determine the normal variation and confounding effects as well as to provide heritability estimates for sMFAP4 to provide a detailed characterization of this biomarker to facilitate further investigations of sMFAP4 levels in liver fibrosis as well as other pathologies with increased ECM turnover.

## Materials and Methods

### Ethics statement

Written informed consent was obtained from all twins and the study was conducted according to the Helsinki declaration. The study was approved by The Regional Scientific Ethical Committee for Southern Denmark, who briefed other regional and the national committees because of the nationwide character: S-VF-19970271. Immunizations in mice were approved specifically by the Danish Animal Experiments Inspectorate and the Animal Ethics Council (license no. 2006/561-1255).

### Buffers and reagents

The buffers and reagents used included Tris-buffered saline (TBS): 140 mM NaCl, 10 mM Tris-HCl, 0.02% (w/v) NaN_2_, pH 7.4; TBS/Tw: TBS containing 0.05% (v/v) Tween 20, pH 7.4; phosphate-buffered saline (PBS): 137 mM NaCl, 3 mM KCl, 8 mM Na_2_HPO_4_, 1.5 mM KH_2_PO_4_, pH 7.4; substrate buffer: 35 mM citric acid, 67 mM Na_2_HPO_4_, pH 5.0; SDS-PAGE sample buffer: 100 mM Tris, 2% SDS, 10% glycerol, 0,0145% bromophenol blue, pH 8.9; and coating buffer: 15 mM Na_2_CO_3,_ 35 mM NaHCO_3_, 0.05% Na-azide, pH 9.6.

### Expression and purification of recombinant human MFAP4

A recombinant protein representing full-length human MFAP4 was expressed in Flp-In^TM^ T-REx^TM^ CHO cells as previously described [5]. In brief, selection for stable integration was achieved by growth in hygromycin B-containing F12 medium (Ham, Glutamax^TM^, Invitrogen). Confluent cells were washed three times in PBS and allowed to grow for 72 h. The supernatant was harvested and centrifuged at 10,000 xg for 20 min at 4°C before filtration using a 0.45 µm filter. Finally, the supernatant was dialyzed against PBS and loaded onto a HisTrap HP column (GE Healthcare). The recombinant protein contained an N-terminal His_6_- and V5-tag, and application of an imidazole gradient eluted the full-length recombinant MFAP4v5His protein (recMFAP4v5His). Similarly, recombinant full-length human MFAP4 without a His_6_- and V5-tag (recMFAP4) was expressed in CHO cells by insertion into the plasmid pcDNA5 FRT Topo and growth in F12 medium (Ham, Glutamax^TM^, Invitrogen) with 1% penicillin*-*streptomycin. The protein-containing supernatant was used for the calibration curve in the ELISA described below. 

### Production and biotinylation of mouse monoclonal anti-MFAP4 antibodies

MFAP4-deficient C57BL6 mice (unpublished data, Schlosser A, 2013) were immunized s.c. with 10 µg purified recMFAP4v5His in 200 µl PBS/Freund’s complete adjuvant for the first round of immunization. At 2-week intervals, three subsequent immunizations were similarly performed using incomplete Freund’s adjuvant. Mice with high antibody titers were selected for splenectomy by ELISA using 96-well Maxisorb microtiter plates (Nunc, Roskilde, Denmark) coated with recMFAP4v5His (1 µg/ml in coating buffer). B cell hybridomas were produced by splenocyte fusion with myeloma cells (ATCC, CRL-2016 and Sp2/mIl-6), and recombinant V5His-coated microtiter plates were used for negative selection. Twenty-one antibody clones were obtained, and monoclonal antibodies were purified using HiTrap Protein G affinity chromatography (GE Healthcare) as previously described by Schlosser et al. [5].

To obtain F(ab’)_2_ antibody fragments, monoclonal antibodies were dialyzed against 0.1 M acetate buffer, pH 4.5. Next, a pepsin (Sigma Aldrich) stock solution (1 mg/ml) was added to a final concentration of 3% (w/w pepsin/antibody) and incubated at 37°C for 16 hours. The mixture was separated by size chromatography on a Superdex 200 column (HiLoad 26/60, GE Healthcare) in TBS to purify the F(ab’)_2_ fragments.

Antibodies were biotinylated by the addition of 166 µl biotin *N*-hydroxysuccinimide ester (Sigma, H1759) (stock of 10 mg/ml biotin dissolved in dimethyl sulfoxide) to 1 ml of antibody (1 mg/ml in PBS, pH 8.5) followed by incubation for 4 hours at room temperature (RT). Excess biotin was removed by dialysis against PBS, followed by TBS.

### Immunoprecipitation

Coupling of monoclonal antibodies HG-HYB 7-5 to cyanogen bromide-activated Sepharose 4B (GE Healthcare) was performed using 10 mg antibody per milliliter of gel as recommended by the manufacturer. Anti-OVA antibody was used as the control antibody (SSI, HYB 099-01). The coupled beads were added to human serum samples diluted 1:10 and left to incubate for 2 hours at RT with gentle agitation. Then, the slurry was washed twice in TBS, 500 mM NaCl, 5 mM CaCl_2_ before elution using 100 mM glycine HCl, pH 2.7.

### SDS-PAGE and Western blotting

Unreduced protein samples were prepared by dilution in SDS-PAGE sample buffer, heating at 100°C for 1 min, and alkylation by the addition of iodoacetamide to a final concentration of 90 mM. Reduced protein samples were diluted in sample buffer containing 60 mM dithiotreitol before heating and alkylation. Protein samples were separated on 4-12% Criterion XT Bis-Tris Gels (Bio-Rad) with a discontinuous buffer system. The separated proteins were electroblotted onto Amersham Hybond-P polyvinylidene difluoride membranes (GE Healthcare). The membranes were incubated overnight at 4°C with one of the monoclonal anti-MFAP4 antibodies at concentrations in the range of 1 – 2 µg/ml in TBS/Tw containing 2.5% (w/v) dry milk. The membranes were subsequently incubated with horseradish-peroxidase-coupled rabbit anti-mouse IgAb (1:10,000, P0206, Dako) for 1 hour at RT. The membranes were washed and developed using ECL Plus Western Blotting Detection Reagents (GE Healthcare), and chemiluminescence was detected with Hyperfilm ECL (GE Healthcare).

### Purification of human MFAP4 from serum

Purification of human MFAP4 from serum was performed by antibody-affinity chromatography using a Fast Performance Liquid Chromatography (ÄKTA-FPLC) system (GE Healthcare). Monoclonal antibody HG-HYB 7-5 was coupled to CNBr-activated Sepharose^TM^ 4B (GE Healthcare) and packed into an XK16 column (GE Healthcare). The washing and binding buffer was PBS/0.5 M NaCl, pH 7.4, and the elution buffer was 3 M MgCl_2_, pH 7.4. Human serum was applied without dilution, and the collected fractions were immediately dialyzed against 10 mM phosphate buffer, pH 6.5, using a Medicell International Ltd dialysis membrane. Anion-exchange chromatography using a Resource^TM^ Q column (pre-packed, 1 ml, GE Healthcare) was then performed with elution in 10 mM phosphate buffer, 1 M NaCl, pH 6.5. Protein purity was analyzed by SDS-PAGE followed by silver staining.

### Gel permeation chromatography

Gel permeation chromatography was performed as previously described [5]. Briefly, a sample volume of 200 µl was applied to an analytical Superose 6 10/300 GL column (GE Healthcare) connected to the ÄKTA-FPLC system using TBS, 10 mM (Ethylenediaminetetraacetic acid) EDTA, 0.05% emulphogen as the eluent at a flow rate of 0.4 ml/min. A standard protein mixture (BioRad, cat.no.151-1901) containing thyroglobulin (670 kDa), thyroglobulin monomer (335 kDa), IgG (158 kDa), ovalbumin (44 kDa), myoglobin (17 kDa), and Vitamin B12 (1.35 kDa) was applied to the column in a separate run.

### Characterization of monoclonal antibodies and selection for assay application

Monoclonal antibodies with reactivity against v5His were excluded from analysis by ELISA chessboard titrations of recMFAP4v5His versus the monoclonal antibodies in two-fold dilutions. Subsequently, competitive analysis was performed to avoid the combination of a pair of antibodies binding to the same epitope. Microtiter plates were coated with recMFAP4v5His (1 µg/ml in PBS), and combinations of the biotinylated antibody (1 µg/ml), and unlabeled antibody (two-fold dilution from 25 µg/ml) were added in TBS/Tw. 

For pairwise optimization, anti-MFAP4 antibodies (1 µg/ml) were used to coat the microtiter plates before incubation with the MFAP4 culture supernatant. The biotinylated antibody was added in chessboard titrations, and the most optimal antibody pair was chosen based on the best signal to noise ratio.

Further ELISA optimization was achieved by an investigation of various coating conditions by including different concentrations of human serum albumin (HSA) in the buffers for the incubation of samples (0.1% and 1% v/v) and by testing the use of pepsin digested F(ab)_2_-anti-MFAP4 antibodies. 

### Measurement of sMFAP4 concentrations by ELISA technique

The sMFAP4 ELISA was performed essentially as described before with minor modifications [14]. Certified 96-well Maxisorb plates (Nunc, Roskilde, Denmark) were coated with 2 µg/ml F(ab)_2_ anti-human MFAP4 IgG (HG-HYB 7-5) by incubation overnight at 4°C in PBS. Each step was performed using 100 µl/well and followed by four rounds of washing in TBS/Tw. TBS/Tw was also used to block for unspecific reactivity by incubating for at least 15 minutes. Plates were stored for several weeks at 4°C before use. Samples, quality controls and standards were diluted in TBS/Tw and allowed to incubate overnight at 4°C. The following morning, biotinylated anti-MFAP4 IgG in TBS/Tw (0.9 µg/ml HG-HYB 7-18) was added and allowed to incubate for one hour at RT on a rocking table. Streptavidin-conjugated horseradish peroxidase (Zymed, Invitrogen) was diluted 1:4,000 in TBS/Tw and incubated for half an hour at RT. Finally, OPD (0.8 mg/ml, Kementec, Taastrup, Denmark) dissolved in 50 mM citric acid (titrated to pH 5.0 with 1 M Na_2_HPO_4_, 0.03% H_2_O_2_ added prior to use) was added and allowed to react for 15 minutes in the dark at RT. Color development was stopped by the addition of 100 µl 1 M H_2_SO_4_, and the plates were read at OD_492_ nm with OD_600_ nm as reference.

The calibration curve was obtained by serial 2-fold dilutions of the culture supernatant from CHO cells expressing the full-length untagged recombinant human MFAP4 (recMFAP4). The pure culture supernatant remains stable for several months when kept at 4°C or -20°C, whereas supernatant diluted in different buffers (TBS/Tw; TBS/Tw, 0.1% albumin; TBS/Tw, 1% albumin) exhibits declining recMFAP4 content upon storage. For this reason, a new serial dilution was prepared for each ELISA run from aliquots of pure culture supernatant, with dilutions ranging from 1:100 to 1:6,400.

Each run included two quality controls prepared from recMFAP4 over-expressing cell culture supernatant using large suspension volumes to minimize dilution error (*Q high* and *Q low*). The controls were adjusted with an aim to obtain an ELISA signal intensity near the boundary of the dynamic range of the assay. All samples were analyzed in duplicate: standard 2-fold dilution calibration-curve samples (as described above), quality control samples, and serum samples.

A 4-parameter logistic method [15] was used to estimate the sample MFAP4 content (U/ml) from the absorbance measurement data (OD_492_). Each ELISA run had to fulfill the following set of criteria to be accepted: 1) the five least dilute samples in the calibration curve had a CV% below 5%; 2) the quality control samples had a CV% below 5%; and 3) the results from more than 10 runs were used to construct a Levy-Jennings plot for each quality control sample with controls only allowed to deviate 2 standard deviations from the mean.

### Assay validation

To document parallelism, several serum and EDTA-plasma samples were analyzed in 2-fold dilutions along with a calibration curve. Estimates of inter-assay variation were calculated for each of the internal controls, including 46 measurements for both *Q high* and *Q low*. All accepted runs from a series of consecutive ELISAs were included in the analysis. The intra-assay variation was determined by the analysis of 2 blood donor samples, each measured 12 times on one ELISA plate. The assay range was estimated graphically from logarithmically transformed titration series of several serum and EDTA-plasma samples.

### Study populations

Samples from blood donors (n = 103, age 19-65, female/male = 56/47) were obtained from the Department of Clinical Immunology, Odense University Hospital, according to the general permission described in the Danish Standards of Transfusion Medicine (TMS ver.3.2.2012, http://tms-online.dk). Other samples used in the assay validation procedure were withdrawn from volunteer individuals working within the laboratory. Danish twins aged 18 to 67 years were identified and enrolled through the Danish Twin Registry as formerly described [16]. Serum MFAP4 measurements from 1,417 twins were available. Exclusion criteria were cardiovascular disease, known diabetes, pregnancy or breastfeeding. The zygosity of the twins was determined using polymorphic DNA-based microsatellite markers [17]. 

### Robustness of sMFAP4 measurements

A total of 10 blood samples were drawn using 2 different types of blood sampling tubes (serum and serum-gel) and 5 different post-venipuncture handling methods (combinations of varying time intervals before centrifugation (30 minutes or 6 hours, 1, 2 or 8 days) and varying time intervals (immediately or 6 hours) and storage temperatures (4 °C or -20 °C) before separation) from 6 healthy volunteers working within the laboratory. Robustness with regard to ten freeze-thawing rounds was investigated using sera from 5 healthy blood donor samples.

### Twin analysis

To approximate a normal distribution, serum MFAP4 measurements were initially logarithmically transformed. Inverse normal plots comparing values of the observed distribution with the corresponding values of the normal distribution exhibited an approximate linear relationship, and the first round of analysis in the mixed effects model described beneath was performed. Using this approach, the significantly important confounding variables were identified and included in box-cox analysis. The theta value obtained from this analysis was used to transform the serum MFAP4 measurements a second time. Inverse normal plots and Shapiro-Wilks test were applied to confirm that a normal distribution could be assumed.

The twin analysis included monozygotic (MZ), dizygotic same-sex (DZ), and dizygotic opposite-sex (OS) twin pairs. The basic theory behind classical twin analysis is based on the concept that monozygotic twins have identical genetic backgrounds, whereas dizygotic twins on average share half of their genes. Additionally, an assumption about equal environment for both monozygotic and dizygotic twins applies. By this assumption, a greater phenotypic variance in DZ twins compared to MZ twins is explained by the greater proportion of genes shared between the latter. In other words, greater phenotypic variance within DZ pairs is to be expected if there is a significant genetic influence on the phenotype studied.

In standard structural equation modeling, the total phenotypic variation is described as the sum of genetic and environmental variation contained within the variance components A, D, C and E [18]. The genetic influences can be split into additive and non-additive genetic components: the additive genetic component, A, represents the sum of the effects of individual alleles that influence the phenotype, whereas the dominance genetic component, D, represents interactions between alleles at the same locus. The environmental influences split into two components as well: the common variation, C, and unique variation, E. This distinction is based on an assumption of the twins having similar environments as they live in the same family (C), but some experiences are unique to the individual sibling (E). Further assumptions generally accepted are the absence of gene-environment interactions, no epistasis (interaction of alleles at different loci), random mating and Hardy-Weinberg equilibrium [19].

The biometrical modeling can be performed as a mixed effects model treating responses for the single twins (family members) as responses of different units nested within the twin pair (family) [19]. The model contains the variance components A, C, D, and E as described above and can be written as an error components model given by the following equation: *y*_*ij*_ = *µ* + *A*_*ij*_ + *D*_*ij*_ + *C*_*ij*_ + *ε_ij_.*

The overall mean is µ, and all components are assumed normally distributed, and assumed mutually independent. Hence, the total variance is equal to the sum of the variance components: 

$$\text{var}\left(y_{ij}\right)\;=\sigma^{2}{}_{\text{A}}+\sigma^{2}{}_{\text{D}}+\sigma^{2}{}_{\text{C}}+\sigma^{2}{}_{\text{E}}.$$

Of the four terms described, a maximum of three can be estimated simultaneously. The method for selecting the best variance structure model followed standard procedures testing nested models compared to full models guided by non-significant P-values and Akaike’s information criterion (AIC). The statistical analyses were performed using Stata ver. 11.2 and R ver. 2.15.1 (www.r-project.org).

## Results

### Production, relative epitope mapping, and species cross-reactivity of monoclonal anti-MFAP4 antibodies

**Figure 1:**
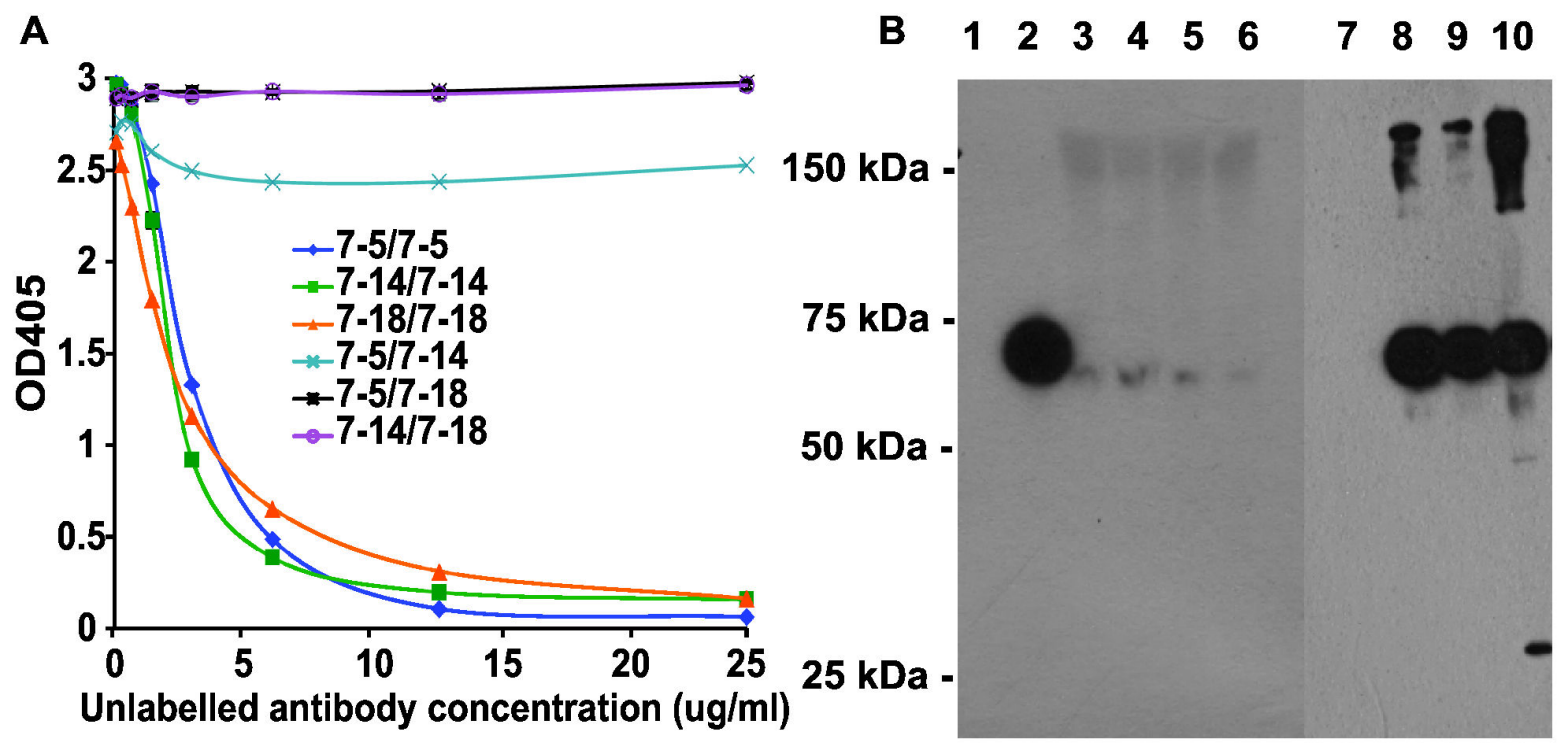
Antibody characterization. **A**. Competitive analysis of monoclonal antibodies against MFAP4. Microtiter plates were coated with recombinant recMFAP4v5his (1 µg/ml). Combinations of labeled and unlabeled antibodies were added using serial dilution of the unlabeled antibody as illustrated on the abscissa. The combinations are described in the legend with the biotinylated antibody (1 µg/ml) before the unlabeled antibody. **B**. Western blots developed using monoclonal HG-HYB 7-5 and visualization by horseradish-peroxidase-coupled rabbit anti-mouse IgG. Lane 1 shows culture supernatant containing recombinant mouse MFAP4 (no band), lane 2 shows CHO-culture supernatant containing human recMFAP4v5His, and lanes 3-6 show four human serum samples diluted 1:10 in sample buffer. Lane 7-10 show the results after immunoprecipitation using Sepharose 4B coupled with monoclonal anti-OVA (HG-HYB 099-01) (lane 7) or with monoclonal anti-MFAP4 (lane 8: HG-HYB 7-5, lane 9:HG-HYB 7-14, lane 10:HG-HYB 7-18).

Recombinant human MFAP4v5His was produced and purified as previously described. The obtained recombinant MFAP4v5His served as the antigen source for the mouse immunizations and, thus, monoclonal antibody production. An initial panel of 21 antibodies binding to recombinant human MFAP4 was included for further analysis. Relative epitope mapping experiments were performed to exclude antibodies with similar epitope reactivity, resulting in a final panel of three antibodies (HG-HYB 7-5, 7-14, 7-18) exhibiting no inhibition of each other (Figure 1A). Two antibodies (HG-HYB 7-14 and 7-18) were found to possess reactivity toward both mouse and human MFAP4, whereas the last antibody (HG-HYB 7-5) only exhibited reactivity against human MFAP4 (Figure 1B, lane 1).

### ELISA optimization and validation

The use of the F(ab)_2_-antibody as the capture antibody was preferred to avoid unspecific reactivity when analyzing human serum sample material, and only HG-Hyb 7-5 proved to be satisfactorily cleaved by pepsin. The choice of the detector antibody HG-HYB 7-18 was based on the best signal to noise ratio. 

**Figure 2:**
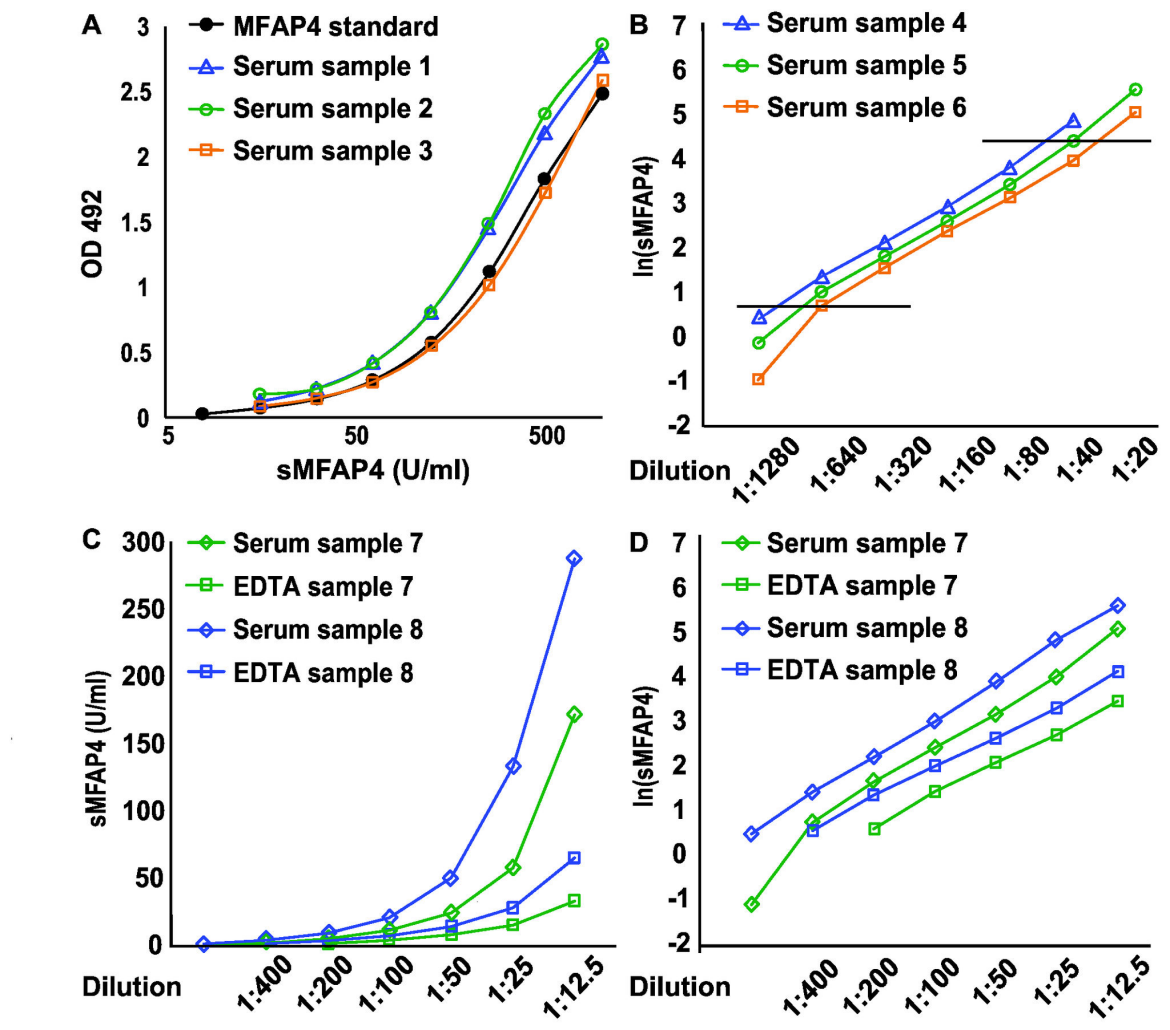
Validation of parallelism and range estimation. **A**. Two-fold dilution curves of sMFAP4 ELISA measurements of samples from 3 representative blood donors illustrate parallelism. The black line represents the standard two-fold dilution curve of culture supernatant from CHO cells expressing recMFAP4 (ranging from 1:100 to 1:6,400). By four-parameter logistic regression, this standard curve is used to estimate the sMFAP4 content in the blood donor samples. **B**. The range of the sMFAP4 ELISA estimated by logarithmically transformed results from two-fold dilution curves similar to A. The results from another set of 3 representative serum samples are shown; the horizontal lines correspond to the highest and lowest acceptable measurements (corresponding to sMFAP4 values of 77.2 U/ml and 1.3 U/ml, respectively). **C**. The difference in sMFAP4 measurement in serum and EDTA-plasma samples is illustrated using samples from 2 healthy blood donors in a two-fold titration. **D**. Parallelism between serum and plasma is obvious after logarithmically transforming the sMFAP4 measurements shown in C. OD: Optical density.

Parallelism in the ELISA detection of recombinant and native MFAP4 was documented by running serial dilutions of donor serum samples along with recMFAP4-overexpressing cell culture supernatants (Figure 2A). The working range of the ELISA was estimated to be 4-75 U/ml by logarithmical transformation of serial dilutions of a number of serum samples (Figure 2B). The final range estimate was chosen to be more conservative than the observed boundaries suggested. Measurements of paired serum and EDTA-plasma samples consistently demonstrated that the EDTA measurements were lower than the corresponding serum results (Figure 2C). Nonetheless, parallelism between the serum and EDTA measurements could be confirmed by serial dilution of several paired samples (Figure 2D). Measurements of samples from 103 healthy blood donors confirmed that sMFAP4 measurements were lower in EDTA-plasma than in serum; the mean sMFAP4 (EDTA-plasma) was 7.9 ± 2.4 (SD) U/ml versus a mean sMFAP4 (serum) of 19.1 ± 5.4 (SD) U/ml. Blandt-Altman analysis revealed a correlation coefficient of 0.80, with sMFAP4 measurements in serum being on average 2.5 ± 1.2 (SD) times higher than the level measured in EDTA-plasma.

The ELISA intra- and inter-assay variations are shown in Table 1. The CV% for the quality controls was 6.6% for *Q high* and 8.7% for *Q low*, representing inter-assay variation. The maximal intra-assay CV% was 4.0%.

**Table 1:** Inter- and intra-assay validation of the MFAP4 ELISA.

	**Inter-assay variation**		**Intra-assay variation**	
	Q high	Q low	Donor sample 1	Donor sample 2
N	42	42	12	12
Mean	53.3	14.1	20.7	33.8
St.dev.	3.55	1.23	0.61	1.35
CV%	6.6	8.7	3.0	4.0

The ELISA used coating with 2 µg/ml F(ab)_2_-HG-HYB 7-5 anti-MFAP4 and detection by 0.9 µg/ml HG-HYB 7-18 anti-MFAP4. The inter-assay variation is calculated for the quality control samples (*Q high*: culture supernatant diluted 1:200, *Q low*: culture supernatant diluted 1:750 in TBS/Tw). Intra-assay variation is calculated based on 12 measurements from two individual samples from healthy blood donors on one plate.

Potential assay interference from biotin and lipids was tested and found negative (data not shown). 

### MFAP4 in human serum

Sera from 4 different individuals were demonstrated to contain MFAP4, as illustrated by Western blotting of sera diluted 1 to 10 (Figure 1B, lanes 2-6). Moreover, all three monoclonal antibodies (HG-HYB 7-5, 7-14, 7-18) were used to immunoprecipitate MFAP4 from human serum samples, and Western blotting demonstrated that all three antibodies were able to precipitate the MFAP4 present in human serum (Figure 1B, lanes 7-10). The blots exhibited bands corresponding to size estimates of 66 kDa using non-reducing conditions. This is the expected size of MFAP4 as determined previously [5,14]. 

**Figure 3:**
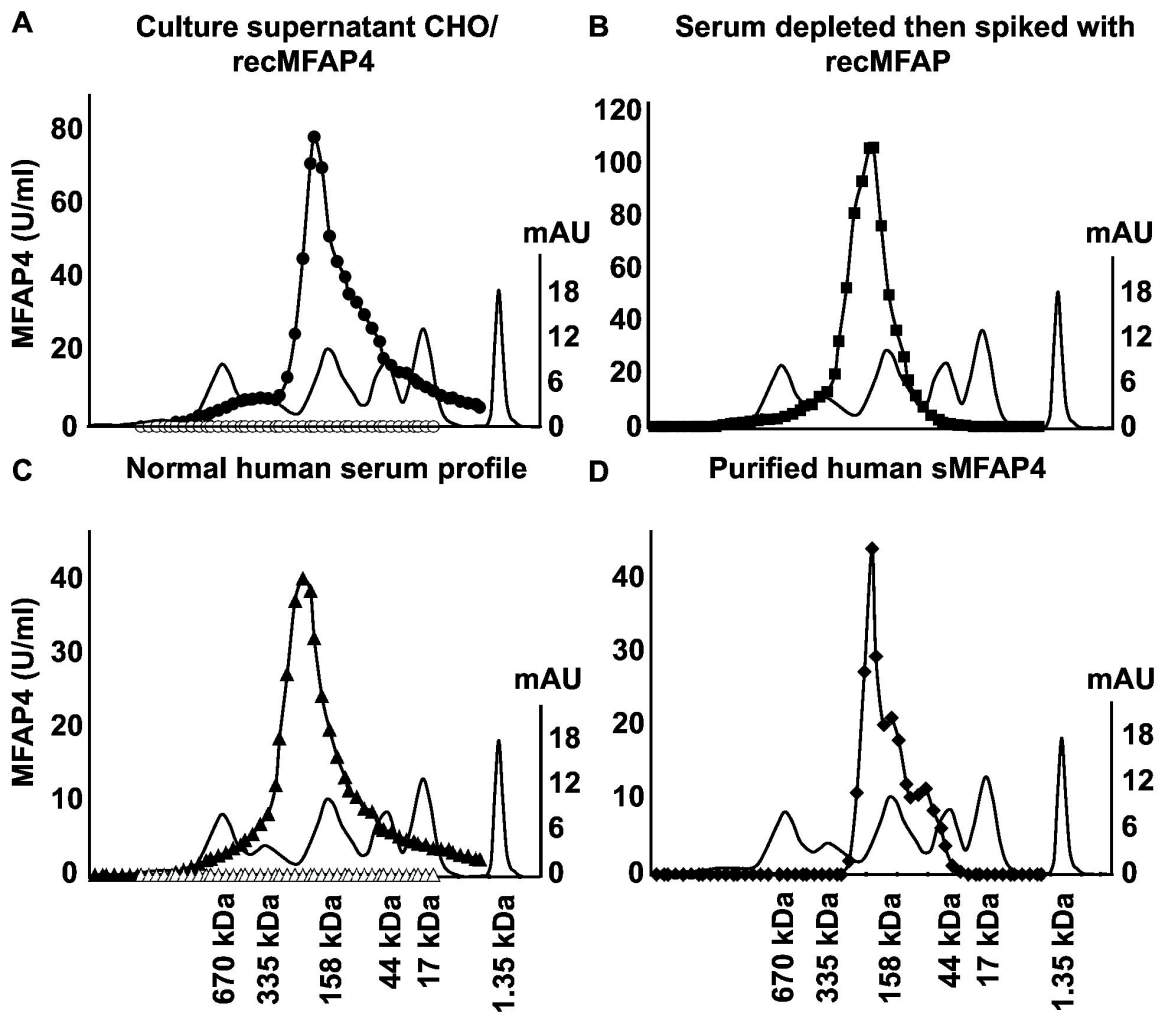
Gel permeation chromatography. Protein elution profiles are shown from the analytical Superose 6 10/300 GL column gel permeation chromatography. The smooth lines in all four graphs (A-D) show the profile of a standard protein mixture containing thyroglobulin (670 kDa), thyroglobulin monomer (335 kDa), IgG (158 kDa), ovalbumin (44 kDa), myoglobin (17 kDa), and vitamin B12 (1.35 kDa). The corresponding values on the right ordinate represent the UV absorbance measure (mAU). All samples were collected in 1-ml fractions analyzed by MFAP4 ELISA as described. The numbers on the left ordinate correspond to the estimated MFAP4 content in U/ml. **A**. Culture supernatant from CHO cells over-expressing recMFAP4 (filled circles) and a negative control of culture supernatant from CHO cells transfected with an empty construct (open circles). **B**. Normal human serum (filled triangles) and normal human serum depleted of MFAP4 (open triangles). **C**. MFAP4-depleted normal human serum reconstituted with recMFAP4 (filled squares). **D**. Affinity-chromatography purified human MFAP4 (filled rhombus).

Gel permeation chromatography was performed to investigate the *in vivo* multimerization of human sMFAP4. Relevant fractions from the chromatographic procedure were analyzed by ELISA (Figure 3). The profiles from the analysis of recMFAP4-overexpressing cell culture supernatant, normal human serum, and reconstituted human serum demonstrated a predominant multimer size of more than 200 kDa (Figure 3A, 3B, 3C). Finally, analysis of the purified native protein was performed, demonstrating two chromatographic peaks possibly corresponding to partly degraded and less multimeric forms of sMFAP4 (Figure 3D). The results confirm the conformation of sMFAP4 in serum to be uniform and multimeric as previously suggested [5] and, moreover, that the antibodies selected for use in the ELISA setup specifically detected this form of sMFAP4.

### Pre-analytical handling

**Figure 4:**
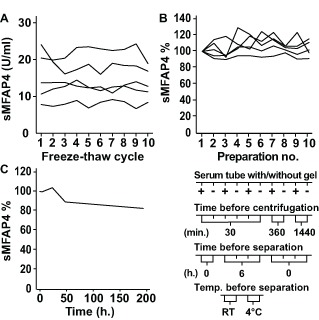
Pre-analytical handling. **A**. Ten aliquots of serum samples from 5 volunteers were subjected to up to ten cycles of thawing and freezing. Each line represents the ELISA results measuring sMFAP4 (U/ml) in aliquots from one single volunteer. The CV% per volunteer ranges from 7.1% to 12.6%. **B**. Eight serum samples from a single, healthy volunteer were left at RT for varying time intervals before centrifugation and separation (1, 5, 24, 48 or 192 hours). The sMFAP4 measurement for the one-hour sample is used as the index sample corresponding to 100%. **C**. Ten serum samples were withdrawn from 5 healthy volunteers using different tube types (serum tubes or serum-gel tubes) and varying temperature and time intervals before centrifugation and separation as indicated below the graph. Each line represents the ELISA results from one single volunteer using preparation number 1 as the index sample corresponding to 100%. The CV% per volunteer for all ten measurements ranges from 3.5% to 9.6%.

Serum MFAP4 measurements were stable up to 10 cycles of freezing and thawing (Figure 4A). The maximal CV% was found to be 12.6%, and the mean CV% was 11.9%. There was no obvious trend of decreasing MFAP4 ELISA detection during the 10 freeze-thaw cycles. Ten samples from five volunteers were used to investigate differences in pre-analytical handling (Figure 4B). Sampling serum in standard tubes with or without gel demonstrated no significant differences in the measured sMFAP4 level. Likewise, no significant differences in sMFAP4 measurements were found when samples were stored for up to 24 hours at 20 °C before being centrifuged or when samples were kept at RT or in the refrigerator for 6 hours before centrifugation. Using all ten results from each individual and each type of preparation resulted in a maximum CV% of 9.6% (Figure 4B). Additionally, in one serum sample from a single high-expressing volunteer, the fall in sMFAP4 content was below 20% after storage for 8 days at RT (Figure 4C). Negligible differences in sMFAP4 measurements were likewise found when analyzing samples kept in the refrigerator (5 °C) compared to samples frozen (-20 °C) after centrifugation (data not shown).

### Confounding variables and heritability of sMFAP4

The study population of 1,417 twins was divided into 288 pairs of monozygotic (MZ) twins, 290 pairs of dizygotic same-sex (DZ-SS) twins and 129 pairs of dizygotic opposite-sex (DZ-OS) twins. The basic characteristics of the twin population have been previously described [20]. This study sample had an almost equal gender representation with a mean age of 37.8 ± 10.9 (SD) years. The mean sMFAP4 in the study population was 18.9 ± 8.4 (SD) U/ml (95% CI: 18.50-19.38, median sMFAP4 17.3 U/ml), which is in accordance with previous results reporting a mean value of 17 ± 5.2 (SD) U/ml in a small, healthy control group of 23 individuals [14]. The distribution of sMFAP4 in the twin population divided by zygosity is shown in Table 2. Spiking of known concentrations of affinity purified recombinant MFAP4 into serum samples was used to estimate that 1U/mL corresponds to 17.6 ng/mL sMFAP4.

**Table 2:** Distribution of sMFAP4 in the twin population divided by zygosity.

**Zygosity**	**N (complete pairs)**	**Mean**	**SD**	**Median**	**Range**
All	1417 (707)	18.94	8.45	17.3	4.1 - 99.7
MZ	578 (288)	19.05	9.07	17.2	4.1 - 99.7
DZ	581 (290)	18.88	8.14	17.4	4.5 - 56.1
OS	258 (129)	18.82	7.68	17.5	7.4 - 59.0

Characteristics of MZ, DZ and OS twins in the study population. Mean, median and range of serum MFAP4 measurements in U/ml. MZ = Monozygotic twins; DZ = Dizygotic twins; OS = Opposite sex twins.

The sMFAP4 distribution was right-skewed, and to approximate a bivariate normal distribution, the data were initially transformed to the logarithmic scale. However, the approximation to normality was not optimal, and a box-cox analysis including initially identified confounding variables was undertaken. This analysis resulted in a theta-value of -0.2 and was used in the remaining analysis.

Using mixed-effect linear regression modeling, the mean value structure was determined to be influenced by age, waist-hip ratio, and cigarette smoking status in interaction with gender. All other measurements, such as height, weight, blood pressure, fasting glucose level, fasting insulin level, and additional measurements relating to the metabolic syndrome, did not exhibit any significant effects concerning the serum MFAP4 level.

**Figure 5:**
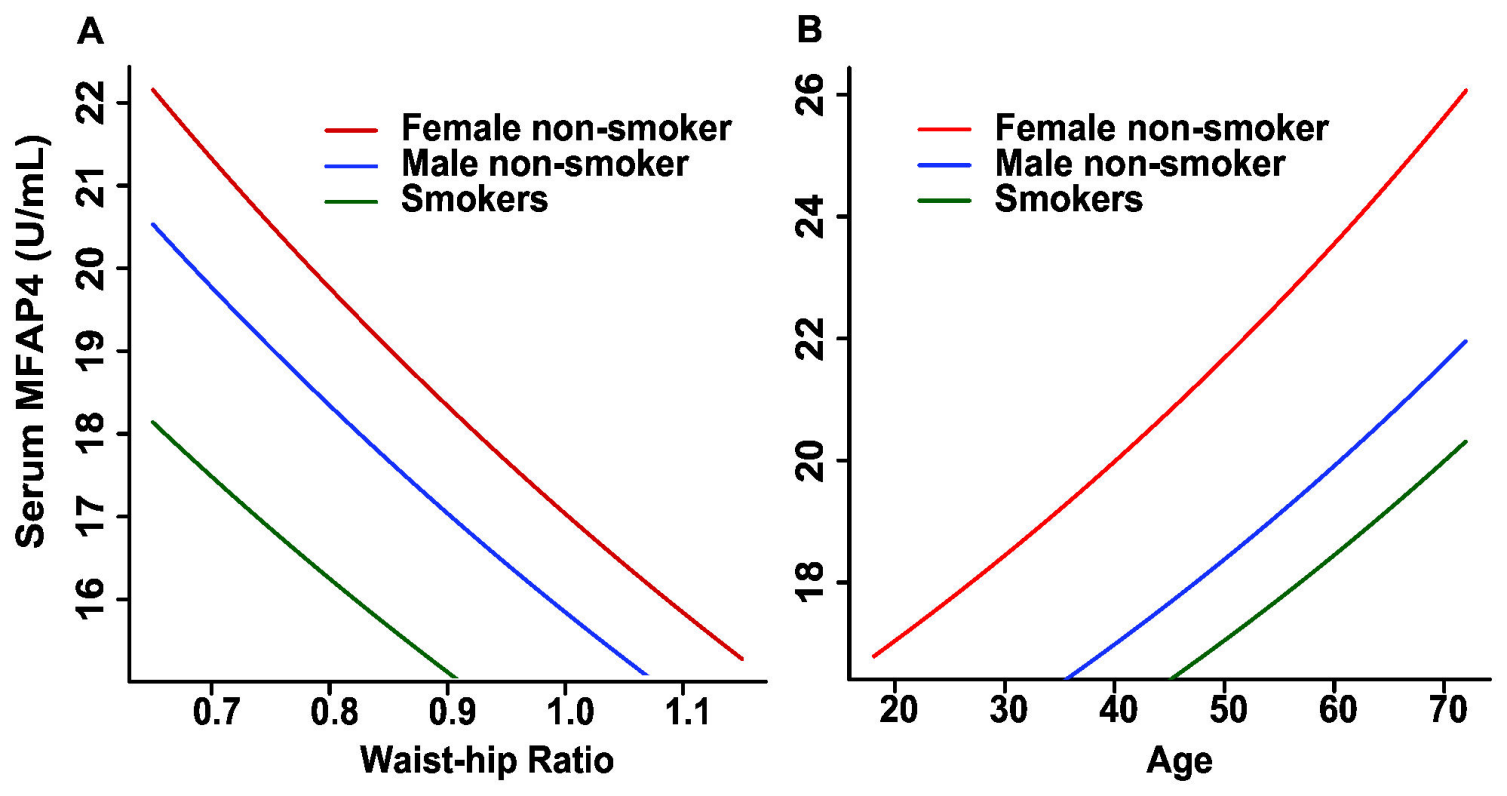
sMFAP4 estimates as a function of age or waist-hip ratio. Serum MFAP4 estimates based on the coefficients in the mean value structure shown in Table 3. **A**. The three graphs report sMFAP4 as a function of age and smoking status (smokers, non-smoking males, and non-smoking females). The mean values of the waist-hip ratio are used in the calculations (females 0.812, males 0.927, and all 0.867). **B**. The three graphs report sMFAP4 as a function of the waist-hip ratio and smoking status (smokers, non-smoking males, and non-smoking females). The mean values of age are used in the calculations (females 37.5, males 38.0, and all 37.7).

Further analysis identified three groups that differed significantly from each other: female non-smokers, male non-smokers, and smokers (Table 3). Male gender decreases the sMFAP4 level compared to females, and cigarette smoking decreases the sMFAP4 level in both genders. However, the effect of cigarette smoking induces a significantly more pronounced reduction in sMFAP4 levels in women. Thus, the analysis demonstrates an apparent elimination of the gender effect due to cigarette smoking. Increasing age is associated with increasing levels of sMFAP4, whereas an increasing waist-hip ratio is associated with decreasing sMFAP4 levels. The effects of the major confounders are illustrated using either a mean value for age or a mean value for the waist-hip ratio to express the sMFAP4 level in the three groups (Figure 5).

**Table 3:** Mean value structure and variance structure.

**Mean value structure**				
Variable	Coefficient	Std. error		P
Gender	-0.000889	0.000120		0.000
Waist-hip ratio	0.0830	0.0165		0.000
Smokers	0.0221	0.00279		0.000
Non-smoking males	0.00871	0.00329		0.008
ε	0.518	0.00134		0.000
**Variance structure**				
Variance-component	Heritability-coefficient		Heritability (%)	
A	0.000403		24.3	
C	0.000433		26.2	
E	0.000818		49.5	

The covariates determined to significantly influence the transformed serum MFAP4 level include age, cigarette smoking, waist-hip ratio, and gender if non-smoking. The corresponding coefficients are presented; the low values are a consequence of the transformation (sMFAP4^0.2). Thus, equations to estimate sMFAP4 levels are as follows:

sMFAP4(smokers) = (0.518-0.000889*age+0.083*waist-hip-ratio+0.0221)^-5,

sMFAP4(non-smoking males) = (0.518-0.000889*age+0.083*waist-hip-ratio+0.0087)^-5,

sMFAP4(non-smoking females) = (0.518-0.000889*age+0.083*waist-hip-ratio)^-5.

The variance components in the ACE-model are presented. The components were determined by the following formula: Heritability (%) = V(x) * 100 / V(total).

The variance structure including additive genetic factors (A), common environmental factors (C), and unique environmental factors (E) provided the best fit for the systemic level of serum MFAP4. The serum MFAP4 level is only moderately influenced by genetic factors, as the genetic heritability (A) is estimated to be a^2^ = h^2^ = 24.3% (Table 3). The model including both additive and non-additive genetic components but leaving out the common environment component (ADE-model) was excluded based on a lower AIC. Likewise, the reduced models AE and CE could be discarded.

## Discussion

The present study describes the technical validation of an ELISA for measuring sMFAP4 in human samples with monoclonal antibodies. The ELISA was used to analyze gel permeation chromatography-separated human serum confirming one multimeric conformation to be the predominant detected form of sMFAP4 in the serum. This result is consistent with previous findings suggesting that sMFAP4 predominantly forms octamers [5]. Pre-analytical handling of samples before the analysis of sMFAP4 content was not a critical factor with respect to temperature or time. The normal variation and heritability were determined, and the confounding variables were identified to be age, waist-hip ratio, and cigarette smoking in interaction with gender. The ACE-model exhibited the best fit and determined the heritability to be 24%. This heritability is relatively low compared to the heritability of additional serum proteins previously measured in the same population eg. surfactant protein D (h^2^ = 0.83) [21]. The low heritability in combination with a relatively limited normal variation supports the possibility that systemic MFAP4 variation within and between patients reflects disease development and progression. 

The ELISA is intended to measure sMFAP4 in clinical blood samples as already done using sera from patients with various degrees of liver fibrosis [14]. For this reason, the one antibody successfully digested by pepsin was used as the capture antibody (HG-HYB 7-5), thereby reducing the risk of problems regarding non-specific binding from rheumatoid factor. The detector antibody producing the best signal-to-noise ratio was HG-HYB 7-18. The inter-assay variation was within reasonable limits, and the general use of Levy-Jennings plots for the process control ensured reliability of the sMFAP4 measurements. The *in vivo* multimerization of human sMFAP4 was investigated by gel permeation chromatography. The predominant form of sMFAP4 in serum appeared to be one major oligomeric conformation. The analysis cannot exclude the presence of larger complexes not detected by the antibodies in use, nor can the existence of sMFAP4 in complex with other proteins be excluded. However, no observations support these possibilities.

The importance of the pre-analytical handling of samples is a topic often ignored despite its vital importance. In a clinical setting, the time from sampling to further processing will inadvertently vary, and the possibility of cooling samples immediately after withdrawal will likewise differ. Therefore, several experiments addressing different aspects of pre-analytical handling were performed. Overall, the findings demonstrated that sMFAP4 is stable in serum for several days and that handling within the first 24 hours resulted in a variation comparable to inter-assay variation. Additionally, the sMFAP4 measurements exhibited no decreasing trend when samples were subjected to up to ten rounds of freezing and thawing. Thus, variation in sMFAP4 measurements due to time and temperature differences when blood samples are handled in standard hospital settings or general practices can be expected to be of limited concern.

The mean systemic sMFAP4 level in the studied twin cohort was found to be 18.9 U/ml (95% CI: 18.5-19.4), and the distribution was non-normal with a median MFAP4 value of 17.3 U/ml. This value is comparable to the observed mean of 17 U/ml in a small control population previously reported [14]. The small difference in the mean sMFAP4 between the two study populations underscores the validity of the determined sMFAP4 estimate of a population mean. Furthermore, the small blood donor cohort exhibited a mean sMFAP4 level of 19.1 U/ml (95% CI: 18.0-20.1) and a median sMFAP4 of 18.3 U/ml.

The twin analysis demonstrated a lower level of sMFAP4 in men compared with women and a lower level of sMFAP4 in smokers compared with non-smokers. Previously, MFAP4 was suggested to act as a bridging protein between the ECM and pulmonary surfactant protein D (SP-D) [8]. Former findings have demonstrated that serum SP-D levels are increased by cigarette smoking and male gender [21]. The expectations for sMFAP4 variation were originally similar, as MFAP4 is likewise highly expressed in the lungs, and MFAP4 may spill over into the circulation from the bronchoalveolar fluid during, e.g., smoking-induced inflammation. However, the level of sMFAP4 expression does not appear to be regulated in the same manner as systemic SP-D, and this difference indicates that the variation in these two systemic biomarkers reflects processes in different tissues or different aspects of tissue turnover.

The measured variable related to body size that was found to be significantly associated with the sMFAP4 level was the waist-hip ratio; neither height nor weight influenced the measure significantly. The relationship was an inverse correlation, as the level of sMFAP4 decreases with increasing waist-hip ratio. A large waist-hip ratio is associated with an increased risk of atherosclerosis and other cardiovascular disease [22]. However, predisposing factors for atherosclerosis, such as blood pressure, were not correlated with sMFAP4 levels in this population-based study, and no conclusions about such relationships were drawn.

Several studies have reported that cigarette smoking accelerates the progression of liver fibrosis in patients with chronic hepatitis C [23], and typical inflammatory biomarkers such as CRP and fibrinogen are elevated in smokers [24]. In contrast, our data demonstrated sMFAP4 to be depressed by current cigarette smoking, indicating that this marker may be regulated differently than inflammatory markers.

The general aim of liver fibrosis biomarker studies is to obtain non-invasive measures to reduce the number of liver biopsies needed, as this is still the accepted gold standard to evaluate fibrosis. However, in spite of numerous studies describing both direct and indirect blood biomarkers of liver fibrosis, none have fulfilled the criteria as an ideal noninvasive marker [25], which would alleviate the need for invasive procedures. The most general problem is misclassification between the fibrosis stages, and to accommodate this misclassification, a range of blood test panels have been developed and described. These panels exhibit reasonably good test characteristics, particularly when combined with transient elastography [26]. Therefore, a putative role for sMFAP4 as a useful biomarker for liver fibrosis and cirrhosis needs further studies in patients who are also being assessed by transient elastography as well as by other recognized biomarkers or combinations thereof.

In conclusion, the described ELISA provides robust measures of the liver fibrosis marker sMFAP4. The heritability and the normal variation of the serum MFAP4 level are relatively low, and the ELISA sMFAP4 measures are stable with regard to pre-analytical variations. The only identified factor affecting the basal systemic level besides age, gender, and cigarette smoking status was the waist-hip ratio. The findings support the biomarker potential of systemic MFAP4, with elevated levels in the serum reflecting pathological processes involving ECM remodeling and degradation.

## References

[1] Zhao Z, Lee CC, Jiralerspong S, Juyal RC, Lu F et al. (1995) The gene for a human microfibril-associated glycoprotein is commonly deleted in Smith-Magenis syndrome patients. Hum Mol Genet 4: 589-597. doi:10.1093/hmg/4.4.589. PubMed: 7633408. 7633408

[2] Kobayashi R, Tashima Y, Masuda H, Shozawa T, Numata Y et al. (1989) Isolation and characterization of a new 36-kDa microfibril-associated glycoprotein from porcine aorta. J Biol Chem 264: 17437-17444. PubMed: 2793866. 2793866

[3] Toyoshima T, Yamashita K, Furuichi H, Shishibori T, Itano T et al. (1999) Ultrastructural distribution of 36-kD microfibril-associated glycoprotein (MAGP-36) in human and bovine tissues. J Histochem Cytochem 47: 1049-1056. doi:10.1177/002215549904700809. PubMed: 10424889. 10424889

[4] Toyoshima T, Ishida T, Nishi N, Kobayashi R, Nakamura T et al. (2008) Differential gene expression of 36-kDa microfibril-associated glycoprotein (MAGP-36/MFAP4) in rat organs. Cell Tissue Res 332: 271-278. doi:10.1007/s00441-008-0587-7. PubMed: 18322703. 18322703

[5] Schlosser A, Thomsen T, Shipley JM, Hein PW, Brasch F et al. (2006) Microfibril-associated protein 4 binds to surfactant protein A (SP-A) and colocalizes with SP-A in the extracellular matrix of the lung. Scand J Immunol 64: 104-116. doi:10.1111/j.1365-3083.2006.01778.x. PubMed: 16867155. 16867155

[6] Hirano E, Fujimoto N, Tajima S, Akiyama M, Ishibashi A et al. (2002) Expression of 36-kDa microfibril-associated glycoprotein (MAGP-36) in human keratinocytes and its localization in skin. J Dermatol Sci 28: 60-67. doi:10.1016/S0923-1811(01)00148-7. PubMed: 11916131. 11916131

[7] Toyoshima T, Nishi N, Kusama H, Kobayashi R, Itano T (2005) 36-kDa microfibril-associated glycoprotein (MAGP-36) is an elastin-binding protein increased in chick aortae during development and growth. Exp Cell Res 307: 224-230. doi:10.1016/j.yexcr.2005.03.005. PubMed: 15922742. 15922742

[8] Lausen M, Lynch N, Schlosser A, Tornoe I, Saekmose SG et al. (1999) Microfibril-associated protein 4 is present in lung washings and binds to the collagen region of lung surfactant protein D. J Biol Chem 274: 32234-32240. doi:10.1074/jbc.274.45.32234. PubMed: 10542261. 10542261

[9] Kasamatsu S, Hachiya A, Fujimura T, Sriwiriyanont P, Haketa K et al. (2011) Essential role of microfibrillar-associated protein 4 in human cutaneous homeostasis and in its photoprotection. Sci Rep 1: 164 PubMed: 22355679. 22355679 10.1038/srep00164 PMC3240987

[10] Martinez-Hernandez A, Amenta PS (1993) The hepatic extracellular matrix. II. Ontogenesis, regeneration and cirrhosis. Virchows Arch A Pathol Anat Histopathol 423: 77-84. doi:10.1007/BF01606580. PubMed: 8212543. 8212543

[11] Moller MJ, Qin Z, Toursarkissian B (2012) Tissue markers in human atherosclerotic carotid artery plaque. Ann Vasc Surg 26: 1160-1165. doi:10.1016/j.avsg.2012.06.008. PubMed: 23068427. 23068427

[12] Roy R, Yang J, Moses MA (2009) Matrix metalloproteinases as novel biomarkers and potential therapeutic targets in human cancer. J Clin Oncol 27: 5287-5297. doi:10.1200/JCO.2009.23.5556. PubMed: 19738110. 19738110 PMC2773480

[13] Lichtinghagen R, Michels D, Haberkorn CI, Arndt B, Bahr M et al. (2001) Matrix metalloproteinase (MMP)-2, MMP-7, and tissue inhibitor of metalloproteinase-1 are closely related to the fibroproliferative process in the liver during chronic hepatitis C. J Hepatol 34: 239-247. doi:10.1016/S0168-8278(00)00037-4. PubMed: 11281552. 11281552

[14] Mölleken C, Sitek B, Henkel C, Poschmann G, Sipos B et al. (2009) Detection of novel biomarkers of liver cirrhosis by proteomic analysis. Hepatology 49: 1257-1266. doi:10.1002/hep.22764. PubMed: 19177598. 19177598 PMC2895500

[15] DeLean A, Munson PJ, Rodbard D (1978) Simultaneous analysis of families of sigmoidal curves: application to bioassay, radioligand assay, and physiological dose-response curves. Am J Physiol 235: E97-102. PubMed: 686171. 686171 10.1152/ajpendo.1978.235.2.E97

[16] Kyvik KO, Green A, Beck-Nielsen H (1995) The new Danish Twin Register: establishment and analysis of twinning rates. Int J Epidemiol 24: 589-596. doi:10.1093/ije/24.3.589. PubMed: 7672901. 7672901

[17] Schousboe K, Visscher PM, Henriksen JE, Hopper JL, Sørensen TI et al. (2003) Twin study of genetic and environmental influences on glucose tolerance and indices of insulin sensitivity and secretion. Diabetologia 46: 1276-1283. doi:10.1007/s00125-003-1165-x. PubMed: 12898014. 12898014

[18] Fisher RA (1918) The correlation between relatives on the supposition of mendelian inheritance. Trans R Soc Edinb 52: 399-433.

[19] Rabe-Hesketh S, Skrondal A, Gjessing HK (2008) Biometrical modeling of twin and family data using standard mixed model software. Biometrics 64: 280-288. doi:10.1111/j.1541-0420.2007.00803.x. PubMed: 17484777. 17484777

[20] Fenger M, Benyamin B, Schousboe K, Sørensen TI, Kyvik KO (2007) Variance decomposition of apolipoproteins and lipids in Danish twins. Atherosclerosis 191: 40-47. doi:10.1016/j.atherosclerosis.2006.04.024. PubMed: 16730014. 16730014

[21] Sørensen GL, Hjelmborg JB, Kyvik KO, Fenger M, Høj A et al. (2006) Genetic and environmental influences of surfactant protein D serum levels. Am J Physiol Lung Cell Mol Physiol 290: L1010-L1017. doi:10.1152/ajplung.00487.2005. PubMed: 16361352. 16361352

[22] Canoy D (2010) Coronary heart disease and body fat distribution. Curr Atheroscler Rep 12: 125-133. doi:10.1007/s11883-010-0092-9. PubMed: 20425248. 20425248

[23] Altamirano J, Bataller R (2010) Cigarette smoking and chronic liver diseases. Gut 59: 1159-1162. doi:10.1136/gut.2008.162453. PubMed: 20650922. 20650922

[24] Yasue H, Hirai N, Mizuno Y, Harada E, Itoh T et al. (2006) Low-grade inflammation, thrombogenicity, and atherogenic lipid profile in cigarette smokers. Circ J 70: 8-13. doi:10.1253/circj.70.8. PubMed: 16377917. 16377917

[25] Duarte-Rojo A, Altamirano JT, Feld JJ (2012) Noninvasive markers of fibrosis: key concepts for improving accuracy in daily clinical practice. Ann Hepatol 11: 426-439. PubMed: 22700624. 22700624

[26] Boursier J, Vergniol J, Sawadogo A, Dakka T, Michalak S et al. (2009) The combination of a blood test and Fibroscan improves the non-invasive diagnosis of liver fibrosis. Liver Int 29: 1507-1515. doi:10.1111/j.1478-3231.2009.02101.x. PubMed: 19725892. 19725892

